# Determination of the Concentration of Silver Atoms in Hydrosol Nanoparticles

**DOI:** 10.3390/nano12183091

**Published:** 2022-09-06

**Authors:** Evgeny Abkhalimov, Vadim Ershov, Boris Ershov

**Affiliations:** Frumkin Institute of Physical Chemistry and Electrochemistry, Russian Academy of Science, Leninsky pr. 31-4, 119071 Moscow, Russia

**Keywords:** extinction coefficient, optical spectroscopy, interband transition, plasmon resonance

## Abstract

In this work, we propose a new method for determining the concentration of silver atoms in hydrosols of nanoparticles (NPs) stabilized with various capping agents. The proposed method is based on the determination of IBT absorption in the UV region (a broad band with a weakly pronounced shoulder at ~250 nm). To determine the extinction coefficient at 250 nm, we synthesized silver nanoparticles with average sizes of 5, 10, and 25 nm, respectively. The prepared nanoparticles were characterized by TEM, HRTEM, electron diffraction, XRD, DLS, and UV–Vis spectroscopy. It has been shown that the absorption characteristics of spherical NPs are not significantly influenced by the hydrosol preparation method and the type of stabilizer used. For particles with a size of 5–25 nm, the molar extinction coefficient of Ag0 atoms was found to be equal to 3500 ± 100 L mol^−1^ cm^−1^ at a wavelength of 250 nm. The results of the theoretical calculations of the molar extinction coefficients for spherical nanoparticles are in good agreement with the experimental values. ICP-MS analysis confirmed the applicability of this method in the concentration range of 5 × 10^−7^–1 × 10^−4^ mol L^−1^.

## 1. Introduction

Nanosized silver has a wide range of uses in various fields, including medicine, agriculture, the production and storage of foodstuffs, industry, electronics, and other fields of human activity, which is due to the unique physical, chemical, and biological properties of silver [1,2,3,4,5,6,7,8]. On the other hand, the uncontrollable dissemination of silver nanoparticles causes their penetration into the living environment, and the toxic properties of silver give rise to serious environmental problems [1,2,9,10,11]. During of 2022, more than 3000 papers dealing with newly discovered properties of silver nanoparticles (Ag NPs) and their fields of application have been published [12], and more than 1000 products based on these particles are present on the market [13]. A topical problem is the determination of the concentration of silver atoms in colloidal dispersions (most frequently in hydrosols) against the background of ionic silver species that are simultaneously present. The simplest and the most promising analytical procedure is the determination of the optical characteristics of Ag NPs by UV–Vis spectroscopy.

Silver nanoparticles can be detected visually by their yellow color, and they exhibit the properties of effective sensors [14,15,16,17,18,19]. The absorption spectrum of spherical Ag NPs, caused by the collective absorption of free electrons in the metal, has a narrow, symmetrical band of localized surface plasmon resonance (LSPR) at approximately 400 nm. This LSPR band is overlapped in the UV range (≤320 nm) by a broad band caused by the interband transition (IBT) of bound electrons in the metal (4d→5sp) [20,21,22,23,24,25]. Ellipsoidal Ag NPs have two plasmon peaks in their absorption spectrum, caused by longitudinal and transverse plasmon resonance. The plasmon absorption of free electrons in Ag NPs is very sensitive, not only to the shape, but also to the state of the particle’s surface. Attempts have been made to use LSPR absorption for determining their nanoparticle concentration [26,27,28,29,30,31]. A systematic method has been proposed for determining the extinction coefficient of monodisperse silver nanoparticles ranging in size from 8 to 100 nm [32]. It can be used to determine the concentration and size of citrate-stabilized nanoparticles from the optical spectrum in the LSPR absorption region. Importantly, this method provides a simple means for calculating the concentrations of citrate-capped silver nanoparticles of a wide size range from their corresponding extinction coefficient. However, LSPR absorption is very sensitive to the state of the particle surface and its shape. The replacement of citrate with another stabilizing agent affects the shape and position of the LSPR band. Therefore, most likely, the method has significant limitations in its application.

In contrast to LSPR absorption, the broad IBT absorption band of the bound electrons of silver particles does not vary noticeably with the particle size, and its intensity is proportional to the number of silver atoms in the nanoparticle [20,25]. In other words, the optical density of the solution at the interband transition wavelength (e.g., 250 nm) proportionally corresponds to the amount of silver particles in the solution in the form of nanoparticles. Analysis of the evolution of the electronic state of Ag NPs over the course of their formation, and its effect on LSPR and IBT absorption, has shown that the estimation of the concentration of silver atoms from the intensity of the interband transition is a promising method [33]. That is, the “relative content” of nanoparticles in a silver hydrosol can be judged from the IBT absorption. For a more accurate determination, it is necessary to determine how the molar extinction coefficient depends on the size of Ag NPs.

This study aims to substantiate the method for determining the concentration of silver atoms in hydrosol nanoparticles from the optical density of the peak of the interband transition of bound electrons in the UV range, as well as to calculate the molar extinction coefficient.

## 2. Materials and Methods

### 2.1. Chemicals and Materials

Silver perchlorate monohydrate (AgClO_4_∙H_2_O, 99%, Acros Organic, Geel, Belgium), potassium oxalate (K_2_C_2_O_4_, special purity grade, 99.9%, Reakhim, Moscow, Russia), Sodium polyphosphate (NaPp, Supelco, Bellefonte, PA, USA), Sodium borohydride (NaBH_4_, 98%, Sigma-Aldrich, Steinheim, Germany), tri sodium citrate dehydrate (Na_3_Cit, 99%, Sigma-Aldrich, Steinheim, Germany), and Polyvinylpyrrolidone (PVP, mol. wt. 40000, Sigma-Aldrich, St. Louise, MO, USA) were used. All solutions used in the experiment were prepared with ultrapure water (18.2 MΩ cm).

### 2.2. Synthesis Procedure

#### 2.2.1. Photochemical Synthesis of Spherical Nanoparticles

The “pure” silver hydrosol containing silver nanoparticles (NPs) and stabilizing carbonate ions was prepared by the reduction of silver ions with oxalate ions under the action of pulsed UV radiation [33,34,35]. First, the solution was deaerated by evacuation. Irradiation was carried out in a special glass vessel equipped with a quartz cell (2–4 mL volume) with an optical path of 5 or 10 mm. The solutions were irradiated with a pulsed xenon lamp at the total radiation flux intensity *I*_UV_ = 6.0 × 10^20^ quanta m^−2^ s^−1^. The light flux from a xenon lamp covers the whole UV and visible regions and is most similar to solar light emission.

#### 2.2.2. Synthesis of Polyphosphate Stabilized Nanoparticles

Polyphosphate stabilized silver nanoparticles were prepared as follows: briefly, a 0.2 mL of 5 × 10^–3^ mol L^−1^ solution of silver perchlorate and 2 mL of 1 × 10^–3^ mol L^−1^ NaPp was mixed with 6.8 mL of ultrapure water. Then, 1 mL of a 1 × 10^−2^ mol L^−1^ solution of sodium borohydride was rapidly added to the resulting mixture. The preparation of Ag nanoparticles lasted for 30 min. After the synthesis, the solution was kept for 24 h for the complete decomposition of the borohydride.

#### 2.2.3. Synthesis of Triangular Nanoplate

The triangular silver nanoprism (TNP) was synthetized by Mirkin’s method [36]. A quantity of 61.7 mL of deionized water, a 1 × 10^–3^ mol L^−1^ solution of AgClO_4_ (10 mL), a 1 × 10^−2^ mol L^−1^ solution of sodium citrate (18 mL), and a 6.1 mol L^−1^ solution of hydrogen peroxide (0.3 mL) were mixed together and thoroughly stirred. Then, 10 mL of a 1 × 10^−2^ mol L^−1^ solution of sodium borohydride was rapidly added to the resulting mixture. The preparation of Ag nanoprisms lasted for 30 min. After the synthesis, the solution was kept for 24 h for the complete decomposition of the borohydride.

#### 2.2.4. Synthesis of PVP-Stabilized Nanoparticles

Polyphosphate-stabilized silver nanoparticles were prepared as follows: 1 mL of a 5 × 10^–3^ mol L^−1^ solution of silver perchlorate and 10 of a 5 × 10^–3^ mol L^−1^ PVP solution were mixed with 34 mL of ultrapure water. Then, 5 mL of a 1 × 10^−2^ mol L^−1^ solution of sodium borohydride was rapidly added to the resulting mixture. After the synthesis, the solution was kept for 24 h for the complete decomposition of the borohydride. The prepared NPs were used to test the procedure for determining the concentration of silver atoms in hydrosols of nanoparticles. Before usage, the NPs were kept in refrigerator at 4 °C.

### 2.3. Instrumentation

#### 2.3.1. Optical Spectroscopy

The optical spectra were measured with a Cary 100 Scan spectrophotometer (Varian Inc., Santa Clara, CA, USA) equipped with a Peltier thermostatic cell at 20 °C. The spectra were recorded in quartz cuvettes with optical path lengths of 5 and 10 mm.

#### 2.3.2. ICP-MS

The concentrations of silver in the initial solution and in the hydrosols were determined by inductively coupled plasma mass-spectroscopy with an Element 2 (Thermo Scientific, Bremen, Germany) spectrometer. The silver concentration was estimated as the average of 10 measurements. To determine the concentration of silver in hydrosols, 1 mL of the sample was dissolved in a 1M solution of nitric acid. Data from the ICP-MS analysis are provided in Table S1.

#### 2.3.3. Microscopy

The nanoparticle size, shape, and polydispersity were determined using a JEM-2100 (JEOL, Tokyo, Japan) transmission electron microscope operating at an accelerating voltage of 200 kV. Samples for TEM analysis were prepared by placing a droplet of a colloidal solution on a carbon-coated copper grid (Ted Pella support grid, carbon type-B, 400 mesh, US) and left to dry completely. TEM images were measured manually using the free software ImageJ (https://imagej.net (accessed on 30 July 2022)) and Gwyddion (http://gwyddion.net (accessed on 19 June 2022)). The histograms were calculated on a minimum of 100 randomly selected NPs.

#### 2.3.4. Dynamic Light Scattering

The hydrodynamic size and the ζ-potential of the silver nanoparticles were determined by dynamic light scattering on a Delsa Nano C instrument (Beckman Coulter, Inc., Brea, CA, USA). The wavelength of the scattered laser radiation was *λ* = 658 nm. Measurements were carried out in a quartz cell with an optical path length of 10 mm. Before starting the measurement, the solution was thermostated at 20 °C.

#### 2.3.5. XRD

The XRD study was performed on a Panalytical X’Pert Pro MPD diffractometer (the Netherlands), equipped with a Cu-Kα X-ray source (*λ* = 1.54184 Å) and an X’celerator detector, operating at the following conditions: voltage: 40 kV; current: 40 mA; range: 30°–85° per 30 min; step size: 0.022°. The crystalline phases were identified using X’Pert High Score Plus Software using the PDF-4 Minerals ICDD database.

### 2.4. Theoretical Spectra Calculation

The theoretical spectra of silver nanoparticles were calculated on the basis of the Mie–Drude theory using the classical BHMIE algorithm for Mie scattering from a sphere [21]. The extinction, absorption, and scattering cross-sections were calculated using MiePlot software (v.4.6.21) [37]. The extinction cross-section was converted to the optical density using Formula (1):

*ε* = (6*Q*_ext_log(e)*c*)/(1000*V*_Ag_π*d*), (1)

where *Q*_ext_ is the extinction effeciency; *c—*silver concentration; *V*_Ag_—molar volume; and *d* —particle size.

We recorded the absorption spectra for spherical monodisperse nanoparticles of sizes in the range 5–50 nm. We used in the calculations the data on the dielectric permittivity of silver, obtained by Babar [38], Hagemann [39], Johnson [40], Palik [41], and Stahrenberg [42]. As for the medium, we took for the dielectric permittivity of water at 20 °C from the IAPWS [43] and Segelstein’s [44] data.

## 3. Results

**Figure 1:**
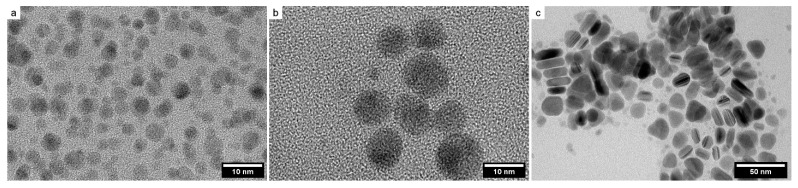
TEM images of polyphosphate (**a**), carbonate (**b**), and plate (**c**) NPs, respectively.

In Figure 1, TEM images of synthesized silver NPs are presented. It can be seen that the NPs synthesized by the polyphosphate and carbonate methods had a spherical shape (Figure 1a,b). Figure 1c shows NPs synthesized by the Mirkin method. They are predominantly in the form of trigonal prisms. In this case, an insignificant number of quasi-spherical particles were observed, which is apparently associated with the partial oxidation of corner atoms during the preparation of a sample for microscopy. NP size distribution histograms are given in the Supplementary Materials (See Figures S1–S3). The hydrodynamic size of the NPs was also determined by the DLS method. The results are presented in Table 1.

**Figure 2:**
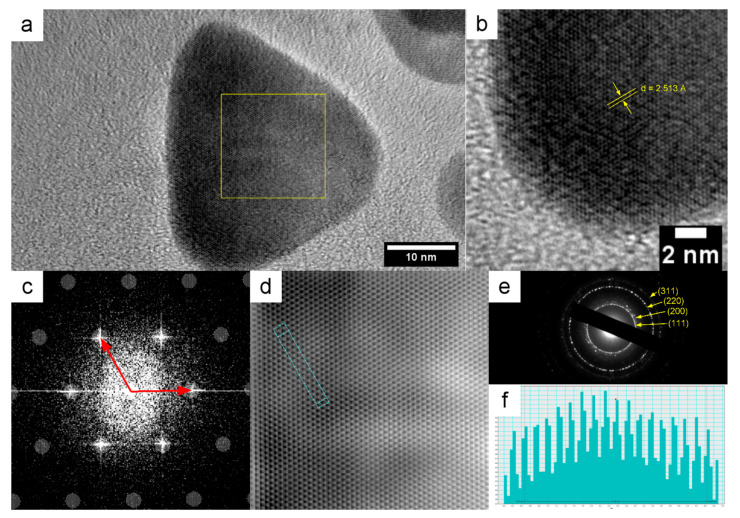
TEM (**a**) and HRTEM (**b**) images of a TNP; (**c**) FFT images of the area highlighted by the square in Figure 2a (**c**); Lattice fringes obtained from Figure 2c by inverse FFT (**d**); Electron diffraction pattern (**e**); Profile of d-spacing of the area highlighted in Figure 2d (**f**).

To confirm that the synthesized silver particles were metallic, the methods of electron diffraction and FFT analysis of the HRTEM images for the measurement of the d-spacing value were used. In Figure 2a,b, HRTEM images of TNP are presented. The d-spacing values of the 111 planes for all NP samples were measured from HRTEM images using FFT analysis. The measured d-spacing values were 2.489, 2.531, and 2.513 Å for polyphosphate, carbonate, and triangular prismatic nanoparticles, respectively. The crystal structure of the NPs was determined by electron diffraction. Figure 2e shows the typical electron diffraction pattern of a silver nanoparticle. In the diffraction pattern, the rings 111, 200, 220, and 311 can be observed. The d-spacing values and the lattice parameters calculated for them have been matched with the face-centered cubic (FCC) structure of silver (JCPDS No. 04-0783), and they confirm the formation of metal NPs. Data for the interplanar distances and the lattice parameters are shown in Table 2. The presence of rings corresponding to Bragg reflections in the electron diffraction pattern established that the silver NPs were polycrystalline. The HRTEM images for polyphasphate and carbonate NPs are presented in the Supplementary Materials (See Figures S4 and S5).

Also, the crystal structure of the NPs was confirmed by X-ray diffraction. The XRD spectrum of 10 nm silver NPs of 2θ = 30°–85° shows the different diffraction peaks at 2θ values of 38.1, 44.3, 64.5, and 77.5 (Figure S6). These peaks matched with the 111, 200, 220, and 311 planes of metallic silver (JCPDS No. 4-0783). The crystallite size was calculated by the Shearer equation from the broadening of the Bragg reflex. Crystallite size was 9.6 nm. X-ray diffraction analysis confirmed the data obtained by the HRTEM method, and they are in good agreement.

Silver-hydrosol-containing metal nanoparticles were prepared by the UV irradiation of a deaerated solution of Ag^+^ ions ((0.2–5) × 10^–4^ mol L^−1^), containing oxalate ions C_2_O_4_^2−^ ((2–5) × 10^–4^ mol L^−1^), at pH ~7.0. The UV light initiates the decomposition of C_2_O_4_^2−^ to generagte CO_2_^–^ radical ions having high a reduction potential (*E*^0^_CO2/CO2–_ = –1.9 V) [45]. These radicals reduce Ag^+^ in the solution volume with the formation of Ag^0^ atoms (*E*^0^_Ag+/Ag0_ = –1.8 V, *k* = 4 × 10^9^ L mol^−1^ s^−1^ [46]):

Ag^+^ + CO_2_^–^ → Ag^0^ + CO_2_ (2)

Silver atoms agglomerate to form metal nanoparticles:

*n*Ag^0^ → Ag*_n_* (3)

and CO_2_ undergoes hydrolysis to form anions stabilizing the colloidal metal particles:

CO_2_ +H_2_O ↔ H^+^ + HCO_3_^–^ ↔ 2H^+^ + CO_3_^2−^ (4)

As a result, a conventionally “pure” silver hydrosol is obtained; it is close in composition to natural fresh water, and, importantly, it contains no stabilizers, reductats, or their transformation products. The mechanism of the formation of the “carbonate” hydrosol and the evolution of the electronic state of the formed nanoparticles and their properties have been studied previously and described in [33,34].

**Figure 3:**
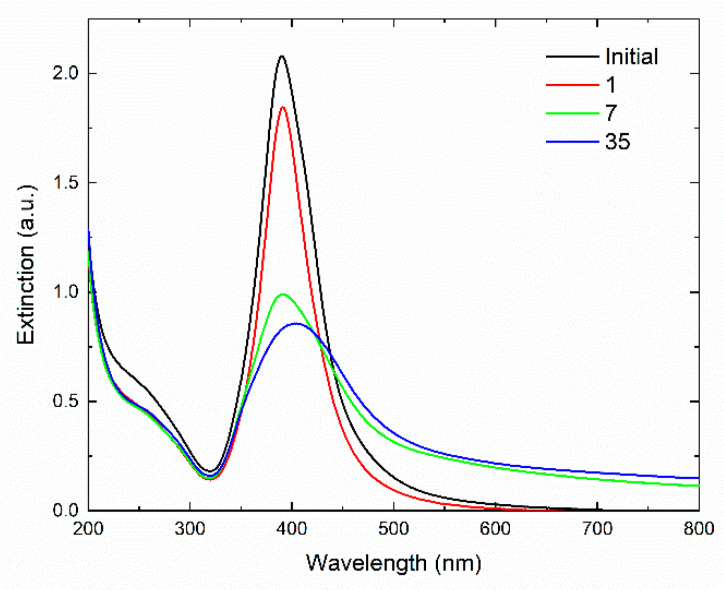
Optical extinction spectrum of silver hydrosol after photochemical reduction of 1.5 × 10^–4^ mol L^−1^ Ag^+^ ions in the presence of 2 × 10^–4^ mol L^−1^ C_2_O_4_^2−^, followed by storage in air for 1, 7 and 35 days, respectively.

Figure 3 shows the absorption spectrum of the “carbonate” silver hydrosol obtained by the complete photochemical reduction of 1.5 × 10^–4^ mol L^−1^ Ag^+^ ions. The spectrum contains a narrow, strong LSPR band at ~400 nm and a broad IBT band with a shoulder at ~250 nm; the IBT band is caused by interband transitions of inner valence electrons. The TEM data show that spherical metal nanoparticles of 9.9 ± 1.3 nm size were formed. The mean size of the colloids, measured by DLS, was 12.2 ± 1.8 nm, and the *ζ*-potential of the hydrosol was –68.3 mV. The negative sign of the potential indicates that the potential-determining layer of the colloid was formed by anions. The HCO_3_^–^ ions formed by photochemical decomposition of oxalate ions were adsorbed on the positively charged surface of the metal core. Together with counterions (H^+^, K^+^, etc.), they formed an electrical double-layer, ensuring electrostatic stabilization of the hydrosol. The high absolute value of the *ζ*-potential shows that the silver colloids were highly stable in the deaerated solution.

Figure 3 also shows that the bands of the excitation of the collective oscillation of free electrons and of the electronic interband absorption in the metal were separated from each other. Namely, the LSPR band is located at approximately 390 nm (or 3.3 eV) or at larger wavelengths, whereas the lower boundary of the 4d→5sp interband electronic transition starts at 320 nm (3.8 eV) and extends toward smaller wavelengths. The absorption of surface plasmons is very sensitive to the state of the nanoparticle surface. Analysis of the LSPR absorption furnishes useful information on the state of the particle and its stabilizing layer, the sorption of molecules with donor and acceptor properties, and other factors that cause changes in the electronic state of the particle surface. In contrast to LSPR, external weak interactions do not noticeably affect the IBT absorption. Figure 3 clearly illustrates this difference. Storage in air significantly influenced the position, shape, and intensity of the LSPR band and did not noticeably affect the IBT absorption. The latter fact indicates that the concentration of silver atoms in the hydrosol remained constant.

**Figure 4:**
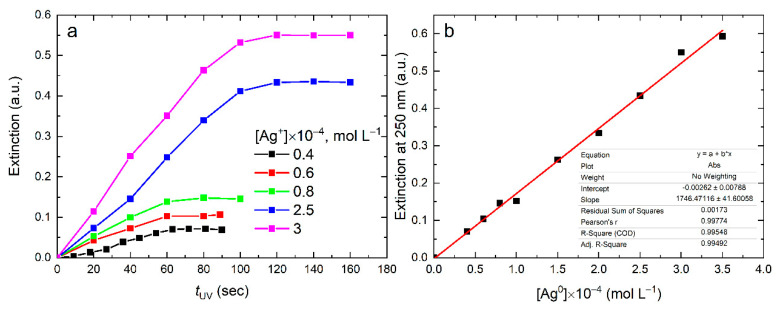
Variation of the absorption of the silver hydrosol during its formation under UV irradiation: variation of the IBT absorption with irradiation time (**a**) and absorption at 250 nm as a function of Ag^0^ concentration (**b**). Solution: Ag^+^ (0.4–3.0) × 10^–4^ mol L^−1^ and C_2_O_4_^2−^ 5 × 10^–4^ mol L^−1^. Optical path length: 5 mm.

In the course of the photochemical reduction of the Ag^+^ ions, the IBT absorption gradually increased and, after UV irradiation for a certain amount of time, reached a constant limiting value corresponding to the complete reduction of the ions and the formation of the colloidal metal (Figure 4a). The lower the initial ions concentration, the shorter the time of their complete reduction.

Upon the complete reduction of Ag^+^ ions in the silver hydrosol, the IBT absorption characterized the concentration of the silver atoms in the nanoparticles. Therefore, it should be expected that the IBT absorption intensity will be linearly proportional to the concentration of the reduced Ag^+^ ions, which is equal to the amount of the formed Ag^0^ atoms. Figure 4b shows the dependence of the absorbance at a wavelength of 250 nm on the concentration of Ag^+^ ions in the solution upon their complete reduction. As can be seen, the linear relationship between the IBT absorption and the concentration of reduced Ag^+^ ions is well-observed in the range from 4 × 10^–5^ to 3.5 × 10^–4^ mol L^−1^. According to the TEM data, the particle size varied within 8–12 nm (error ±1.5 nm) with an increase in the concentration of Ag^+^ ions. The molar extinction coefficient of silver atoms in the nanoparticles, calculated in accordance with the Bouguer–Lambert–Beer law, appeared to be equal to *ε*_250_ = 3493 ± 83 L mol^−1^ cm^−1^.

**Figure 5:**
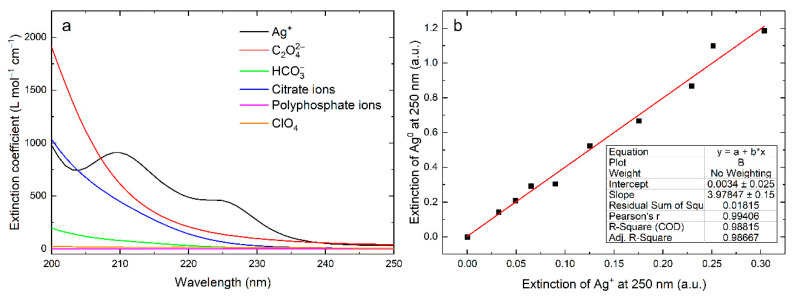
Absorption spectra of Ag^+^ ions and C_2_O_4_^2−^, HCO_3_^–^, citrate, and polyphosphate anions (**a**); relationship between the IBT absorbance (*λ*_max_ = 250 nm) and the absorbance of Ag^+^ ions (*λ*_max_ = 210 nm) (**b**).

Figure 5a shows the extinction spectra of solution components (in L mol^−1^ cm^−1^ units) after photolytic decomposition of oxalate ions C_2_O_4_^2−^ with the complete reduction of Ag^+^ ions and the formation of bicarbonate anions HCO_3_^–^. A comparison of the spectra of Ag^+^, C_2_O_4_^2−^, and HCO_3_^–^ ions with the spectrum of silver nanoparticles at the same reactant concentrations shows that these ions do not noticeably affect the absorption of the colloidal metal. The IBT absorption of Ag^0^ atoms is not noticeably complicated by the absorption of other species, which allows for the measurement of their concentrations in the “carbonate” silver hydrosol at a wavelength of ≥215 nm with good accuracy.

Figure 5b illustrates the relationship between the IBT absorbance (Ag^0^ atoms) at 250 nm and the absorbance of Ag+ ions in the initial solution (*λ*_max_ = 210 nm), which transformed into silver atoms upon reduction. As can be seen, a strict linear relationship can be observed between these absorbances. The slope of the dependence is equal to the *ε*_250_(Ag^0^)/*ε*_210_(Ag^+^) ratio. From the known value of *ε*_210_(Ag^+^) = 901 ± 16 L mol^−1^ cm^−1^ (Figure 5a), we find *ε*_250_(Ag^0^) = 3586 ± 64 L mol^−1^ cm^−1^, which coincides with the value calculated above. Thus, we can conclude that the *ε*_250_ for the carbonate silver hydrosol with spherical particles of ~10 nm size is 3500 ± 100 L mol^−1^ cm^−1^.

**Figure 6:**
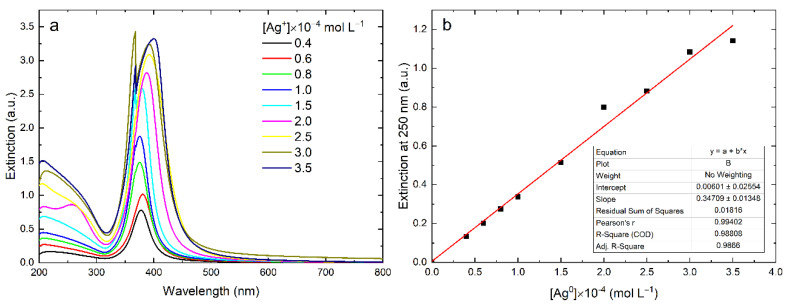
Variation of absorption of the silver hydrosol during its formation by the reduction of Ag^+^ with NaBH_4_ in the presence of NaPp (**a**); IBT absorption at 250 nm as a function of Ag^0^ concentration (**b**). Solution: Ag^+^ (0.4–3.5) × 10^–4^ mol L^−1^ and NaPp 2 × 10^–4^ mol L^−1^ (**b**). Optical path length: 10 mm.

Figure 5 and Figure 6 show the dependences of the IBT on the concentrations of reduced Ag^+^ ions (Ag^0^ atoms formed) for other silver hydrosols. These dependences are similar to the above-considered dependence for the carbonate hydrosol. Figure 6 illustrates the dependence for spherical nanoparticles of a 4.3 ± 0.7 nm size, obtained by the reduction of Ag+ ions with borohydride and stabilized with polyphosphate. As can be seen, there is good linear dependence on the concentration of Ag^0^ atoms, allowing the calculation of *ε*_250_ = 3471 ± 135 L mol^−1^ cm^−1^.

Thus, it can be concluded that, for silver hydrosols with spherical particles of a size from 4 to 12 nm, prepared by different methods using different stabilizing additives, ε_250_ is within 3500 ± 100 L mol^−1^ cm^−1^.

**Figure 7:**
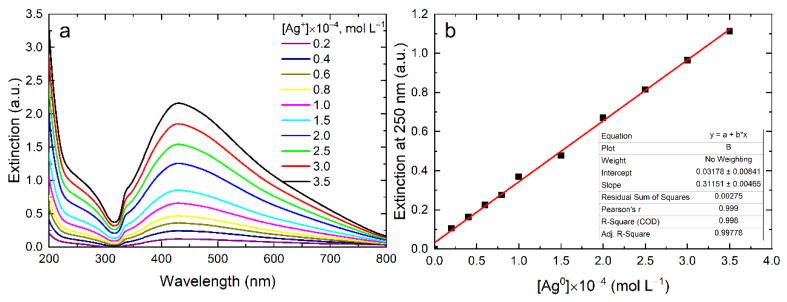
Variation of absorption of the silver hydrosol during its formation by the reduction of Ag^+^ with NaBH_4_ in the presence of sodium citrate and hydrogen peroxide (**a**); IBT absorption at 250 nm as a function of Ag^0^ concentration (**b**). Solution: Ag^+^ (0.4–3.5) × 10^–4^ mol L^−1^ and citrate 1.8 × 10^–3^ mol L^−1^. Optical path length: 10 mm.

Figure 7 shows the dependence of the IBT absorption on the concentration of Ag^0^ atoms for trigonal silver nanoplates with the face size of 23 nm and thickness of 10 nm. The molar extinction coefficient at 250 nm was calculated: *ε*_250_ = 3115 ± 47 L mol^−1^ cm^−1^.

A comparison of the *ε*_250_ for the silver nanoprisms and for the spherical particles shows that, most likely, the molar extinction coefficient tends to decrease when becoming to prismatic particles.

## 4. Discussion

Taking into account the absorption of the individual solution components whose photolysis yields a carbonate silver hydrosol (Figure 5a) and the mechanism of photochemical transformations, we calculated the IBT absorption spectrum of the nanoparticles in the extinction units, L mol^−1^ cm^−1^.

**Figure 8:**
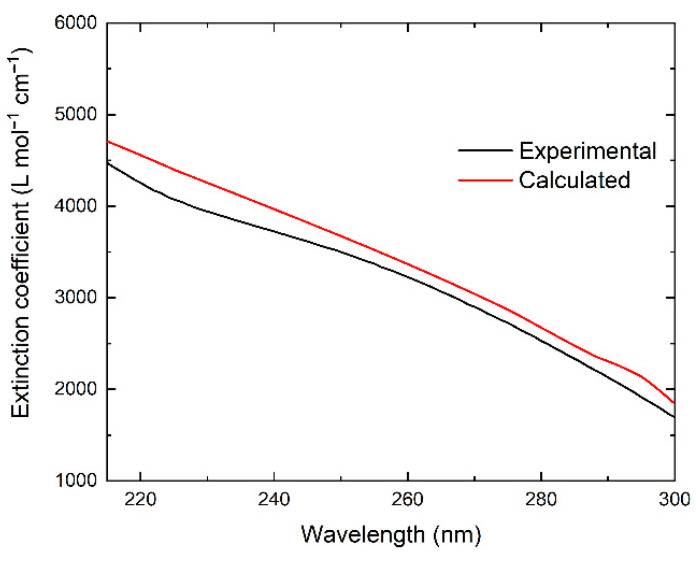
IBT absorption of silver nanoparticles in carbonate hydrosol (black line: experiment; red line: calculated from the dielectric permittivity of silver [39]).

Figure 8 shows the IBT absorption of silver nanoparticles in carbonate hydrosol (red line) and calculated from the dielectric permittivity of silver (black line). To calculate this spectrum, the data on the permittivity of silver obtained earlier by Hagemann were used [39]. The spectrum was calculated for particles of 10 nm with a dispersion of 10%. This spectrum is a broad band with a smeared, ill-defined shoulder at ~250 nm. The band ascends in the short-wave direction and gives way to the LSPR absorption at wavelengths of ≥300 nm. The calculated extinction coefficients at different wavelengths are superimposed on the experimental IBT absorption spectrum. As can be seen, the theory quite adequately describes the dependence of *ε* on *λ*. A clear trend can be observed: a smooth increase in *ε* from approximately 2000 L mol^−1^ cm^−1^ at a wavelength of 300 nm to 3600 L mol^−1^ cm^−1^ at 250 nm and then to approximately 4750 L mol^−1^ cm^−1^ at 215 nm.

**Figure 9:**
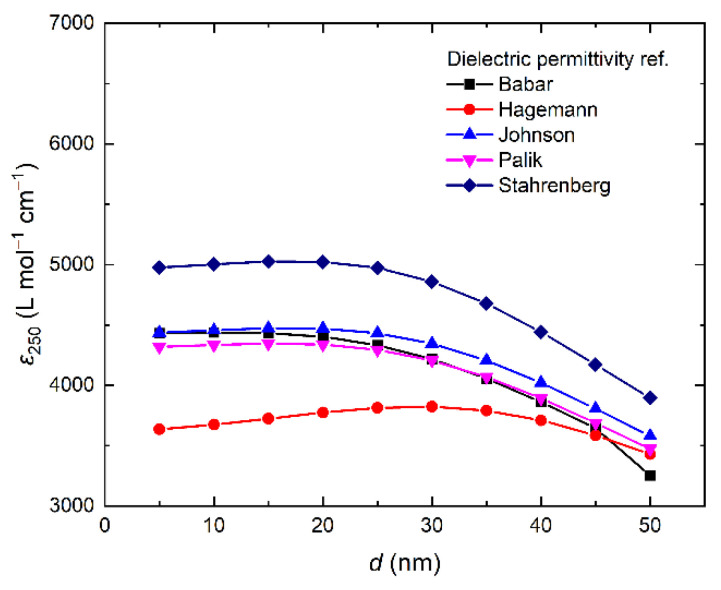
Molar extinction coefficient of Ag^0^ at 250 nm (IBT absorption) as a function of nanoparticle size.

Figure 9 shows the dependences of the molar extinction coefficient of Ag^0^ atoms at 250 nm for nanoparticles with sizes in the range 5–50 nm, calculated using various data on the dielectric permittivity for monodisperse silver nanoparticles in aqueous solution.

Analysis of the dependences obtained shows that the results of the theoretical calculations reasonably agree with the experimental molar extinction coefficient for nanoparticles of approximately 10 nm in size, equal to 3500 ± 100 L mol^−1^ cm^−1^. For example, the Stahrenberg model gives the value of approximately 5000 L mol^−1^ cm^−1^, the Babar, Johnson, and Palik models give approximately equal values of about 4500 L mol^−1^ cm^−1^, and, finally, the Hagemann model gives the value of 3600 L mol^−1^ cm^−1^. That is, in the latter case, the theoretical and experimental values virtually coincide. The theoretical dependence of ε on the nanoparticle size shows that the coefficient varies only slightly for spherical particles of sizes in the range 5–25 nm. With a further increase in the size to 50 nm, *ε* smoothly decreases by approximately 5–20%.

Table 3 summarizes the data on the values of ε250 that we calculated for various silver hydrosols from the figures given in the corresponding works. As can be seen, the majority of the *ε*_250_ values for the different hydrosols, containing spherical nanoparticles, fall into the range of *ε*_250_ determined in this study: 3500 ± 100 L mol^−1^ cm^−1^.

In order to confirm the adequacy of the proposed method for determining the concentration of silver atoms in hydrosols with other stabilizing agents, we synthesized PVP-stabilized silver NPs. The concentration of silver atoms determined by our proposed method was compared with the concentration determined by the ICP-MS method. The obtained data are presented in Table 4. The concentration of silver atoms determined by ICP-MS and by the IBT absorption are in good agreement in the concentration range of 5 × 10^–7^–1 × 10^–4^ mol L^−1^. It should be noted that, when calculating the concentrations of Ag^0^ atoms in different hydrosols, it is, of course, necessary to take into account the possible superposition of the absorption of impurities (reductant, stabilizer, reaction products) on the IBT absorption to improve the analytic accuracy.

## 5. Conclusions

The results of our studies confirm the possibility of determining the concentration of silver atoms in hydrosol nanoparticles by interband transition absorption in the UV range. For particles of 5–25 nm in size, the molar extinction coefficient of Ag^0^ atoms at 250 nm was calculated: 3500 ± 100 mol^−1^ L cm^−1^. It should be emphasized that the hydrosol preparation procedure and, actually, the hydrosol composition only slightly influence the characteristics of the IBT absorption. That is, this absorption is virtually insensitive to the state of the silver nanoparticle surface. This fact allows the developed procedure for determining Ag^0^ atoms in nanoparticles to be considered as versatile and applicable to hydrosols of different origins.

## Figures and Tables

**Table 1:** The characteristics of silver NPs obtained from TEM and DLS technique.

Type of NPs	Surfactant	*d*_TEM_ (nm)	*d*_DLS_ (nm)	*ζ*-Potential (mV)
Sphere	Carbonate	9.9 ± 1.3	12.2 ± 1.8	–68.3
Sphere	Polyphosphate	4.3 ± 0.7	6.5 ± 1.3	–56.8
TNP	Citrate	22.8 ± 4.7; 9.8 ± 1.2	-	–63.9

**Table 2:** Comparison of interplanar spacing as determined by electron diffraction of the Ag NPs.

Sample	d-Spacing (Å)/Miller Indices (hkl)				Lattice Parameter (nm)
	111	200	220	311	
Standard Ag *	2.35	2.04	1.443	1.23	4.079
Polyphosphate	2.49	2.11	-	-	4.266
Carbonate	2.35	2.06	1.44	1.23	4.085
TNP	2.50	2.02	1.44	1.24	4.184

* JCPDS No. 4-0783.

**Table 3:** Comparison of the extinction coefficients (*ε*) obtained in this work and those calculated from the data of other works.

Type of NPs	Surfactant	[Ag] × 10^–4^, mol L^−1^	Extinction at 250 nm	*ε*, L mole^−1^ cm^−1^	*d*, nm	Ref.
Sphere	HCO_3_^–^	0.4–3.0	0.07–0.6 *	3493 ± 83	8–12	This work
Sphere	NaPp	0.4–3.5	0.13–1.14	3471 ± 135	4.3 ± 0.7	This work
TNP	Citrate	0.2–3.5	0.1–1.1	3115 ± 47	Length 30; Thickness 8	This work
Sphere	Benzotriazole	0.5	0.15	3000	40–50	[47]
Sphere	Grape seed extract	1	0.375	3750 **	3–14	[48]
Quasi-spherical	PANa	2	0.754	3770 **	4–25	[49]
Sphere	Chitosane	1.47	0.25 *	3400	13–20	[50]
Sphere	N-(3,5-bis (trifluoromethyl) phenyl)-2-(4-chlorophenyl) hydrazine carbothioamide (ThAm)	0.38	0.13	3420	11	[51]
Sphere	At carbon dots	1.15	0.38	3300	7 nm	[52]

* Optical path length: 5 mm; ** Blank spectra were not subtracted.

**Table 4:** Comparison of concentration of PVP-AgNP measured by ICP-MS and UV–Vis.

[Ag], µg L^−1^	Measured by ICP-MS (ppb)	Calculated by UV–Vis (ppb)	Error (%)
50	61	70	14.4
100	113	126	11.8
500	525	500	4.7
1000	987	1051	6.5
5000	5170	5030	2.7
10,000	10,464	10,142	3.1

## Data Availability

Not applicable.

## Supplementary Materials

The following supporting information can be downloaded at: https://www.mdpi.com/article/10.3390/nano12183091/s1: Figure S1: Size distribution of Pp silver NPs; Figure S2: Size distribution of carbonate silver NPs; Figure S3: Size distribution of silver nanoplates; Figure S4: TEM (a) and HRTEM (b) images of polyphosphate NPs; FFT images of the area highlighted by the square in Figure S4a (c); Lattice fringes obtained from Figure S4c by inverse FFT (d); Profile of d-spacing of the area highlighted in Figure S4d (e); Figure S5: TEM (a) and HRTEM (b) images of carbonate NPs; FFT images of the area highlighted by the square Figure S5a (c); Lattice fringes obtained from Figure S5c by inverse FFT (d); Profile of d-spacing of the area highlighted in Figure S5d (e); Figure S6. XRD pattern of carbonate NPs.
