# Supplementary material for: Determination of the Concentration of Silver Atoms in Hydrosol Nanoparticles
Source: Nanomaterials (Basel). 2022 Sep 6;12(18):3091. doi: 10.3390/nano12183091 (PMC9504487; doi:10.3390/nano12183091)
Supplement: Supplementary file 1 [file nanomaterials-12-03091-s001.zip › nanomaterials-1890069-supplementary.pdf]

Supplementary materials

Determination of the Concentration of Silver Atoms in Hydrosol Nanoparticles

Evgeny Abkhalimov *, Vadim Ershov and Boris Ershov

Frumkin Institute of Physical Chemistry and Electrochemistry, Russian Academy of Science, Leninsky pr. 31-4, 119071 Moscow, Russia
* Correspondence: abkhalimov@ipc.rssi.ru

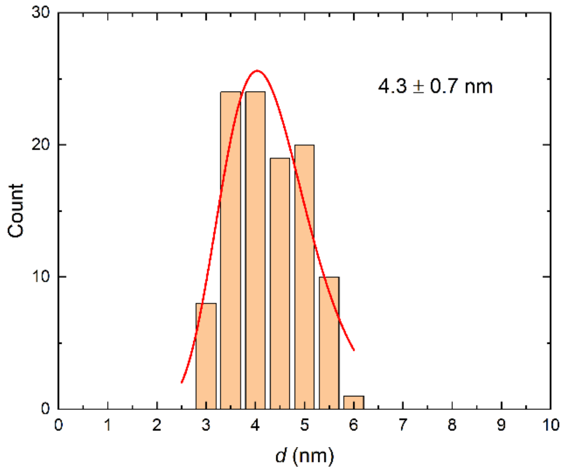

Figure S1. Size distribution of Pp silver NPs.

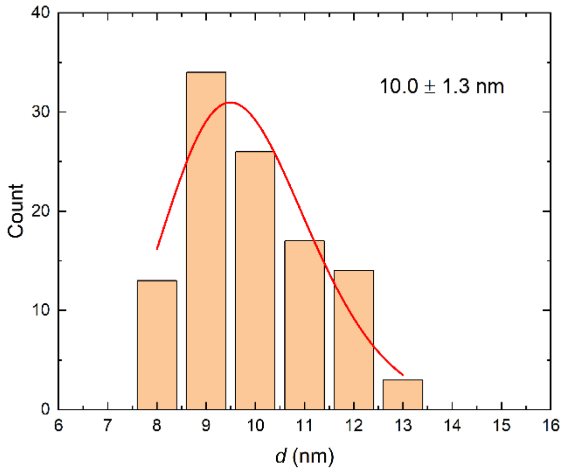

Figure S2. Size distribution of carbonate silver NPs.

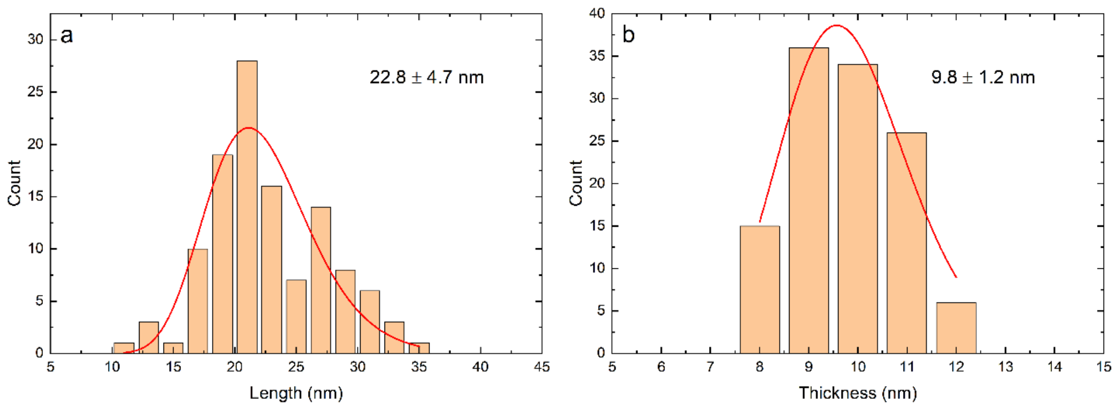

Figure S3. Size distribution of silver nanoplates.

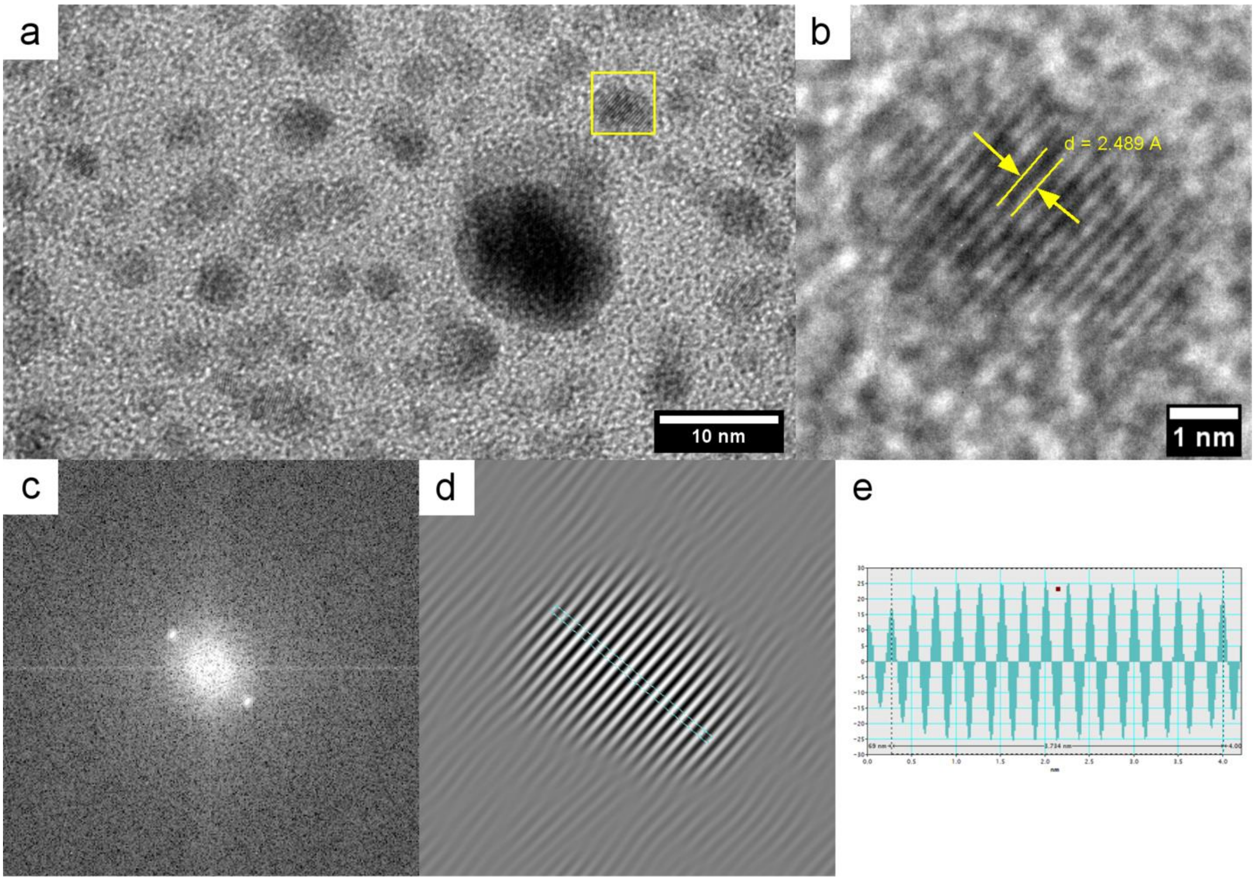

Figure S4. TEM (a) and HRTEM (b) images of polyphosphate NPs; (c) FFT images of the area highlighted by the square in 4a (c); Lattice fringes obtained from 4c by inverse FFT (d); Profile of d-spacing of the area highlighted in 4d (e)

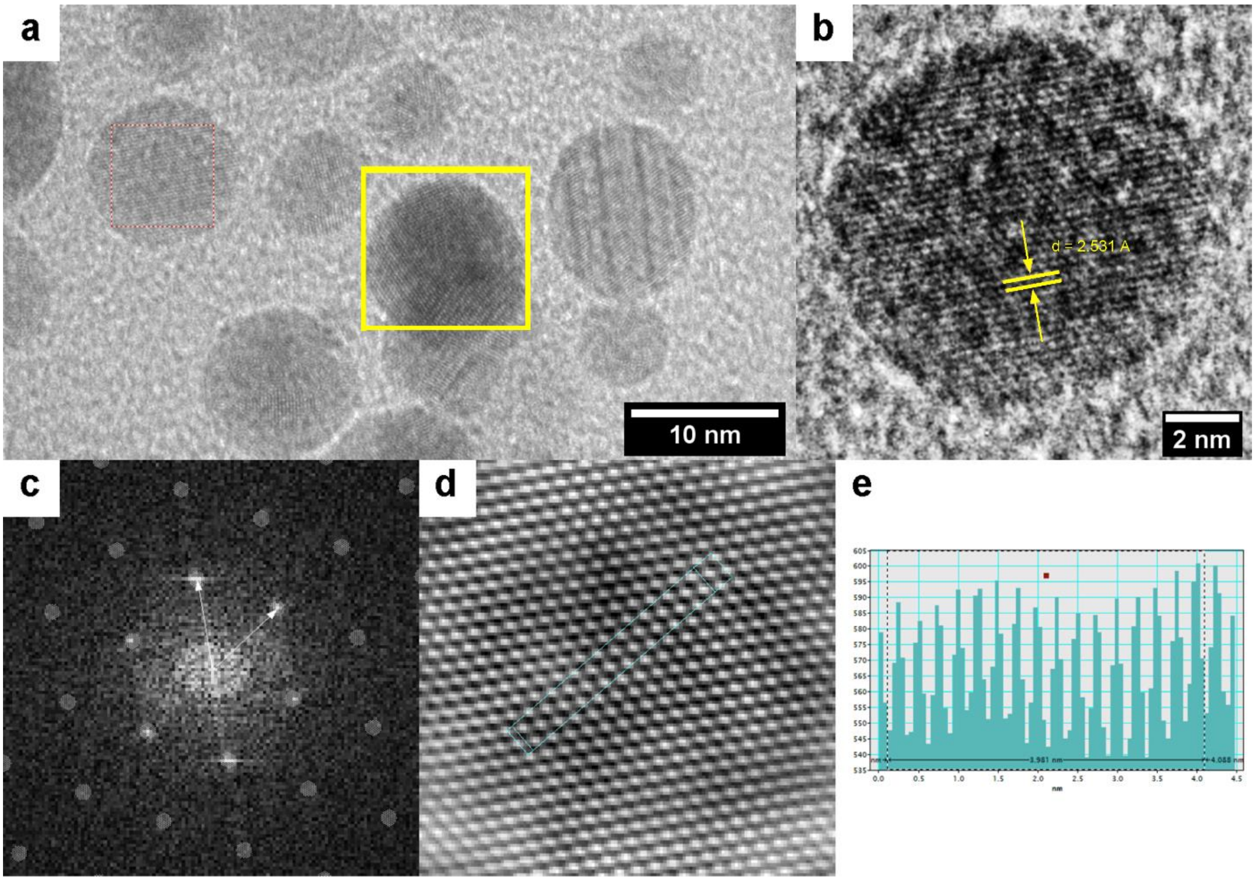

Figure S5. TEM (a) and HRTEM (b) images of carbonate NPs; (c) FFT images of the area highlighted by the square in 5a (c); Lattice fringes obtained from 5c by inverse FFT (d); Profile of d-spacing of the area highlighted in 5d (e).

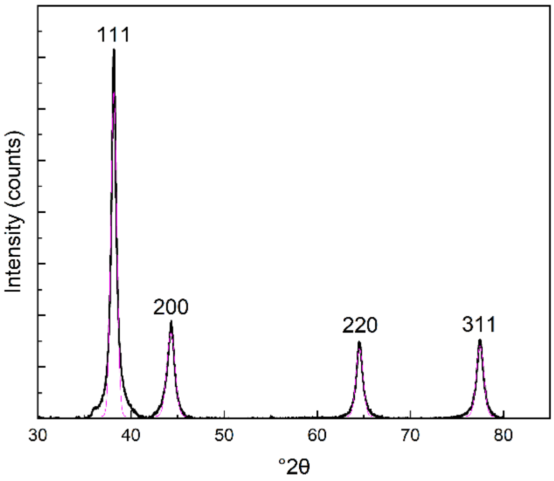

Figure S6. XRD pattern of carbonate NPs.
